# A versatile method for dynamically controlled patterning of small populations of epithelial cells on substrates via non-contact piezoelectric inkjet printing

**DOI:** 10.1371/journal.pone.0176079

**Published:** 2017-04-26

**Authors:** Kristen B. Lee, Laimonas Kelbauskas, Alan Brunner, Deirdre R. Meldrum

**Affiliations:** Biodesign Institute, Arizona State University, Tempe, AZ, United States of America; Osaka Shiritsu Daigaku, JAPAN

## Abstract

Intercellular interactions play a central role at the tissue and whole organism level modulating key cellular functions in normal and disease states. Studies of cell-cell communications are challenging due to ensemble averaging effects brought about by intrinsic heterogeneity in cellular function which requires such studies to be conducted with small populations of cells. Most of the current methods for producing and studying such small cell populations are complex to implement and require skilled personnel limiting their widespread utility in biomedical research labs. We present a simple and rapid method to produce small populations with varying size of epithelial cells (10–50 cells/population) with high-throughput (~ 1 population/second) on flat surfaces via patterning of extracellular matrix (ECM) proteins and random seeding of cells. We demonstrate that despite inherent limitations of non-contact, drop-on-demand piezoelectric inkjet printing for protein patterning, varying mixtures of ECM proteins can be deposited with high reproducibility and level of control on glass substrates using a set of dynamically adjustable optimized deposition parameters. We demonstrate high consistency for the number of cells per population (~1 cell standard error of mean), the population’s size (~0.2 coefficient of variation) and shape, as well as accurate spatial placement of and distance between colonies of a panel of metaplastic and dysplastic esophageal epithelial cells with differing adhesion and motility characteristics. The number of cells per colony, colony size and shape can be varied by dynamically varying the amount of ECM proteins deposited per spatial location and the number of spatial locations on the substrate. The method is applicable to a broad range of biological and biomedical studies including cell-cell communications, cellular microenvironment, migration, and stimulus response.

## Introduction

Communication among cells of the same or different types at the tissue or whole organism level has been long recognized as an important factor in normal and disease states. At a tissue level, cellular function is inherently linked to cell-cell communications. More specifically, the microenvironment and cell-cell interactions have been demonstrated to play a central role in carcinogenesis and development of cancer with manifestations in modulating metastatic potential [1–4]. Despite its widely recognized role and significance, studies of intercellular interactions and their functional relevance remain challenging mainly due to technical limitations of the current experimental approaches [5]. Intrinsic cellular heterogeneity in vivo prevents a detailed insight into the functional role of cellular interactions by obscuring effects caused by cellular communication via ensemble averaging in bulk cell experimental assays. Bulk cell assays generally consisting of 10^5^ to 10^7^ cells are limited to the analysis of population-level average values and completely hide details associated with heterogeneity of cells [6, 7]. Consequently, cellular interaction events taking place among small sub-populations of cells, yet potentially having a profound effect on the survival of the entire population [8], can remain undetected within a bulk sample.

A number of different approaches and techniques have been developed for micropatterning of single cells and small colonies of cells, which can be divided into three main classes: stencil printing, photolithography, and inkjet printing. Stencil printing is based on the creation of cell adhesion islands on an otherwise cell-repellent substrate by using microfabricated stencils to deposit cell adhesion material in the desired areas on the substrate [9–11]. Photolithographic methods rely on UV photoactivation of biomaterials through a high precision mask, which creates areas of interest with differential adhesion properties [12, 13]. Both types of approaches require complex microfabrication equipment and expert skill which has prevented their widespread use in biomedical research laboratories. In this regard, inkjet printing which is based on drop-on-demand non-contact deposition of sub-nL volumes of liquid, offers several distinct advantages over the other technologies [14–17]. First, it can be implemented using commercial inkjet printers or dedicated research-grade platforms without the need to access complex microfabrication equipment. Second, the method is unmatched in throughput and the ability to dynamically control deposited liquid volume and spot size. Two main technologies are used for inkjet printing: thermal and piezoelectric. While thermal inkjet printing is a less expensive alternative, it is limited by the high transient temperatures in the print head that can adversely affect biomaterials and cells. Piezoelectric inkjet printing offers the advantage of not relying on temperature increase, but on mechanical pressure pulse generation instead that leads to droplet release from the print head. However, despite its previous use for biomolecule patterning [14, 17–19], non-contact printing of proteins remains challenging mainly due to the specifics associated with surface tension, fluid viscosity, and buffer rheology properties of the protein mixtures. This leads to a variety of issues, such as missed spots, spot-to-spot variation and sample carryover [20, 21]. While the generation of cell colonies with 350 μm diameter has been demonstrated using a commercial inkjet printer [19], the colony size in the study was fixed and limited by the printer specifications. Matsusaki et al. reported a method for inkjet printing mixtures of fibronectin/gelatin and live cells for 3D tissue-mimicking multicellular structures of varying size [18]. While powerful as a tool for creating multi-layered cell structures, it still involves physical printing of live cells which may cause adverse effects in cell health. Furthermore, deposition of only one fixed formulation of fibronectin and gelatin mixture was demonstrated, potentially limiting the applicability of the method to cell types with differing adhesion and growth properties.

We present a simple non-invasive, yet powerful approach that enables the creation of small (tens of cells) populations of mammalian cells using a variety of ECM proteins mixtures with varying formulations. While the method builds upon the previous techniques of piezoelectric inkjet printing, it is advantageous in that it utilizes an advanced liquid deposition instrument that enables fine tuning of the deposition parameters (pulse shape and length). We demonstrate that by selecting appropriate liquid deposition parameters, mixtures of ECM proteins can be reliably deposited for generating patterns of ECM proteins on substrates with varying shape and size with high throughput (~1 population/second). In contrast to other published approaches, our method is non-invasive as it does not require cell printing and yet offers a high degree of control over the cell number (~1 cell standard error of mean), colony size, and custom spatial patterns. The method is based on preferential binding of cells to regions with cell adhesion-promoting properties on a planar substrate, followed by random seeding of cells, with subsequent removal of excess cells by washing. The adhesion regions are created by dispensing under a tightly controlled set of deposition parameters of 40–100 pL volume droplets of cell adhesion-promoting molecules, e.g. extracellular matrix protein, on a substrate. We developed and optimized a custom set of droplet dispensing parameters that enables deposition of 40–50 pL volumes of fluids containing different mixtures of ECM proteins. The droplet pattern can be customized to produce different spatial arrangements of cell populations on the substrate enabling a high level of control over distances between populations, e.g. for studies including intercellular interaction and migration. For validation purposes we used a panel of four immortalized Barrett’s esophageal epithelium cell lines that represent different stages (metaplasia and various sub-stages of dysplasia) of premalignant progression to esophageal adenocarcinoma with markedly different physiological, morphological, adhesion, and gene expression profiles. The main reason for choosing these four cell lines was to demonstrate the ability of our approach to pattern cells with different physiological and adhesion properties.

This study demonstrates increased uniformity, robustness, reliability and reproducibility of the approach as compared with other techniques for creating small populations of cells. The approach is simple to implement, yet is versatile to be used as a biomedical research tool in cell-cell interaction, migration, and microenvironmental studies. We report on experimental results regarding the number of cells per population, precision and reproducibility of the method.

## Materials and methods

### Substrate preparation and deposition of extracellular matrix (ECM) proteins

Fused silica substrates, 8x8 mm, were sonicated for 15 minutes in a 1% micro-90 solution (Catalog# Z281506, Sigma-Aldrich, St. Louis, MO) and then re-sonicated in an ultrasonic bath (Branson 5510R-DTH, Branson Ultrasonics Corp., Danbury, CT) for 15 minutes in deionized ultrapure water (Purelab Option S-R 7–15, ELGA, Woodridge, IL) to remove any particles or organic material. The substrates were then allowed to air-dry in a laminar flow hood for 24 hours.

The ECM proteins stock mixture was prepared by combining 19.5 μL of poly-L-lysine (PLL, Catalog# P4707, Sigma-Aldrich), 3.0 μL of laminin (Catalog# 114956-81-9, Sigma-Aldrich), and 7.5 μL of fibronectin (Catalog# F1141-1MG, Sigma-Aldrich). The formulation of the mixture has been determined experimentally via testing cell adhesion of four different esophageal epithelial cell lines (CP-A, CP-B, CP-C, and CP-D) representing different stages of premalignant progression in Barrett’s esophagus (BE) [22, 23] as a function of the amounts of the three ECM components. Subnanoliter droplets of the ECM protein mixture were deposited on the substrates using an automated contactless liquid dispensing robot (Rainmaker au301, Engineering Arts LLC, Phoenix, AZ). The Rainmaker is capable of creating the desired patterns of droplets in under 1 minute per substrate, wherein a picoliter volume of liquid was patterned per droplet [6]. For deposition, 30 μL of PLL stock solution was added to the ECM mixture to produce a Supermix B/2 mixture with reduced viscosity to facilitate dispensing. Without dilution with PLL we observed rapid fouling or clogging of the micropipette tip by the ECM stock mixture. As opposed to adding water, adding PLL to the mixture decreased the viscosity of the mixture, yet allowed more ECM material to be deposited on the substrate, overall. This patterning technique allowed for cell populations to be arranged in custom spatial patterns. Optical compatibility of the substrates enabled easy, direct visualization of the patterned cells with conventional light microscopy for cell counting and/or morphology studies. In this study we used the non-contact dispenser to deposit 5x5 arrays of droplets of the ECM proteins mixture. Deposition was performed using a tapered micropipette with a 24 μm opening at the tip. The piezoelectric actuator was driven with an amplitude of 25 volts, and a trapezoidal waveform of 1/4/18 μs. These parameters were determined experimentally to be optimal to achieve the desired deposition volume and the spatial confinement of the ECM on the substrates. The fluid deposition parameters, i.e. voltage, pulse shape and duration, have been determined empirically through trial-and-error and using our earlier knowledge of deposition dynamics using other liquids with varying rheology. All depositions on the substrates were made after first aspirating 2.5 μL of Supermix B/2 into the tip, followed by dispensing on a total of 4 chips in a single run, i.e. without re-loading the tip, and following one of the desired deposition patterns. One or two drops were dispensed onto each desired location on the substrate using a “stop-and-drop” dispense protocol. The protocol was created to stop the tip before depositing Supermix B/2 at a desired location on the substrate, as opposed to “on-the-fly” deposition without stopping the tip. All depositions were performed in a relative humidity of 60%. A visual inspection of the quality of the deposition was performed periodically on each chip during the dispensing step using a built-in camera mounted on the Rainmaker to assure droplet deposition was occurring and on target. After each deposition was finished, the remaining reagent was flushed out of the tip in a flowing stream of deionized (DI) water by pushing 150 μL of DI water through the tip using a syringe pump. This prepared the tip to aspirate a new bolus of the mixture once the appropriate coordinates of the next batch of chips had been transferred to the robot. It was important to keep the lag time between aspiration and dispense operations and between successive dispense operations on different chips to a minimum to prevent fouling of the tip. Also critical to successful dispensing was proper maintenance of back pressure by adjusting the level of the water reservoir to remain slightly positive at all times. The deposition of a 5x5 pattern of the ECM mixture took 25–30 seconds. Once all chips in a batch had been dispensed, they were kept in a covered petri plate and stored at 4°C until used.

### Cell culture

The immortalized metaplastic and dysplastic human Barrett’s esophagus cells (CP-A, CP-B, CP-C, and CP-D cell lines) [22] were cultured in T75 tissue culture flasks (Corning, Corning, NY) to approximately 80% confluence at 37°C, under 5% CO2 atmosphere in cell culture flasks using GIBCO® Keratinocyte SFM cell growth medium (Invitrogen, Carlsbad, CA), supplemented with hEGF (Peprotech, Rocky Hill, NJ) at 2.5 μg/500 mL, BPE (bovine pituitary extract) at 25 mg/500 mL and penicillin/streptomycin solution (Invitrogen) at 100 units/100 μg/mL. Prior to seeding, cells were detached from the flask bottom using a 0.05% trypsin-EDTA solution and concentrated to 10^5^ cells/mL in the cell culture medium. For counting purposes, the cell nuclei were stained with the Hoechst 33343 nuclear DNA stain. Cell viability was determined prior to each experiment using an automated cell counter (Countess, Invitrogen) and was 95–99%.

### Cell preparation and viability assessment

Immediately prior to cell seeding, the patterned substrates were removed from the refrigerator and placed into a dry petri dish. The cell seeding procedure was started by pipetting 50 μL of cell culture medium containing 500 ± 50 cells onto the surface of the substrate, covering the entire substrate and forming a dome-like structure. The substrate, once removed from the refrigerator equilibrates to room temperature. Also, the warmed media increases the substrate rapidly so that it is no longer 4°C by the time the cells settle onto the substrate. At the time of cell seeding the substrate is approximately room temperature and the media containing the cells is still approximately 37°C. The substrate was then placed into a petri dish and incubated at 37°C, 5% CO_2_ for 15 minutes to allow cells to adhere to the patterned areas on the substrate. Afterwards, the substrates were rinsed with warm PBS using a pipette to remove floating cells and weakly attached cells outside the patterned regions. The washing step was performed while observing the substrate under the microscope. For cell counting and cell viability assessment, the substrates were imaged under the microscope using the brightfield and fluorescence modes, respectively. Cell viability after seeding was determined using the CalceinAM/Sytox Orange live/dead assay (Invitrogen, Carlsbad, CA).

### Statistical analysis

Statistical data analysis and significance testing was performed using OriginPro™ (version 9.0, OriginLab Corp., Northampton, MA) software. A two-way ANOVA statistical significance test with Tukey means comparison at p = 0.05 was used to determine the significance of the observed differences in average population sizes.

## Results

**Fig 1:**
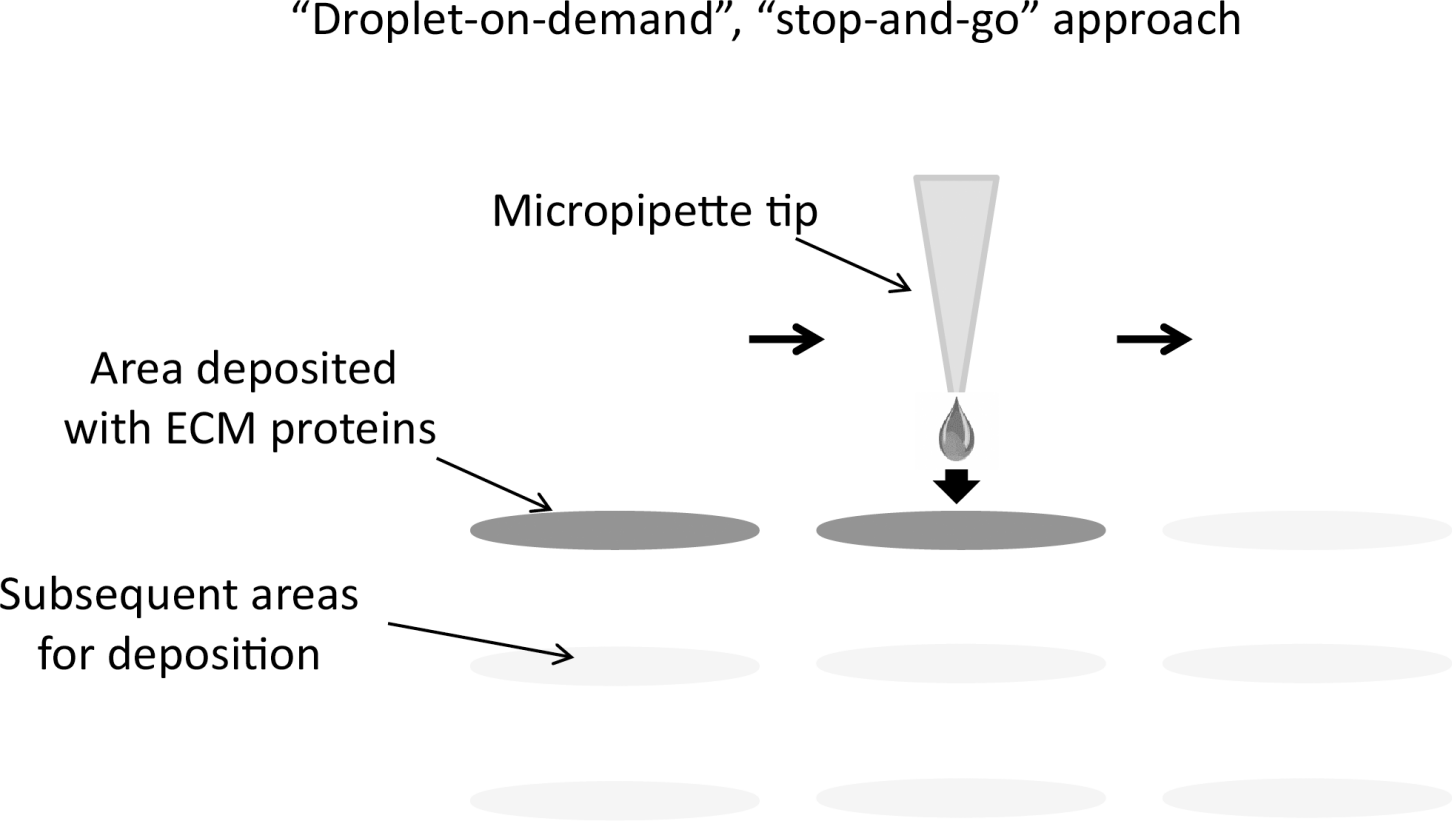
Conceptual approach of the cell micropatterning method used in this study. Subnanoliter volumes of an ECM proteins mixture are deposited on a flat substrate with pre-defined custom spatial patterns. The amount of the deposited mixture as well as its spatial location can be controlled precisely via customizable dispensing parameters for flexibility in controlling the population size and proximity to other adjacent populations.

The approach for patterning cells is to take a substrate, create regions with preferential cell adhesion to incur differential site-specific cell retention, and remove cells from untreated areas on the substrate via fluid flow. Differential cell adhesion is obtained by depositing small volumes of a cell adhesion-promoting material in a desired spatial pattern on the surface of a flat substrate (Fig 1) followed by cell seeding. ECM dispense parameters were optimized to consistently generate discrete droplets that were traveling at 2–3 meters/second at a distance of 400 μm from the nozzle opening. That is the approximate distance that the substrate was located from the nozzle face of the dispense head.

While applied voltage amplitude is a major factor in determining the size of generated droplets, and greater amplitude generally produces larger droplets, it is not the only critical dispense parameter that requires consideration. Since the main physical force keeping the ECM solution (or any liquid) pinned in the nozzle opening at a 90 degree angle to the face of the nozzle is surface tension, it is this surface tension which must be overcome to eject a volume of fluid with sufficient velocity to break free of both the fluid column and the glass of the nozzle. This roughly determines the minimum applied voltage amplitude required to generate discrete droplets. There is also a maximum voltage amplitude beyond which the surface tension pinning the fluid at the nozzle opening is exceeded, causing air to be pulled into the dispense head, which interrupts the fluid path in the nozzle. This fluid path must remain continuous for the dispense head to operate successfully.

The shape of the electronic waveform applied to the piezo-powered dispense head is also a major factor requiring optimization to consistently generate discrete droplets. While more aqueous liquids easily and continuously form discrete droplets by application of a cosine wave-shaped pulse, higher viscosity fluids such as the ECM solution used in this study require a modified electronic waveform to be applied. To maintain the fluid interface at the nozzle opening it is necessary to minimize the transfer of force from the piezo to the fluid during the initial stage of energizing the piezo when it physically contracts. This is achieved by shortening the duration of this portion of the waveform relative to a standard cosine waveform. Modifying the length of time that the piezo physically expands directly affects how quickly the force is transferred to the fluid column and thus to the fluid at the nozzle opening. Too short a duration does not allow enough time for a droplet to be ejected, while longer than optimal duration can cause more fluid than desired to be dispensed.

**Fig 2:**
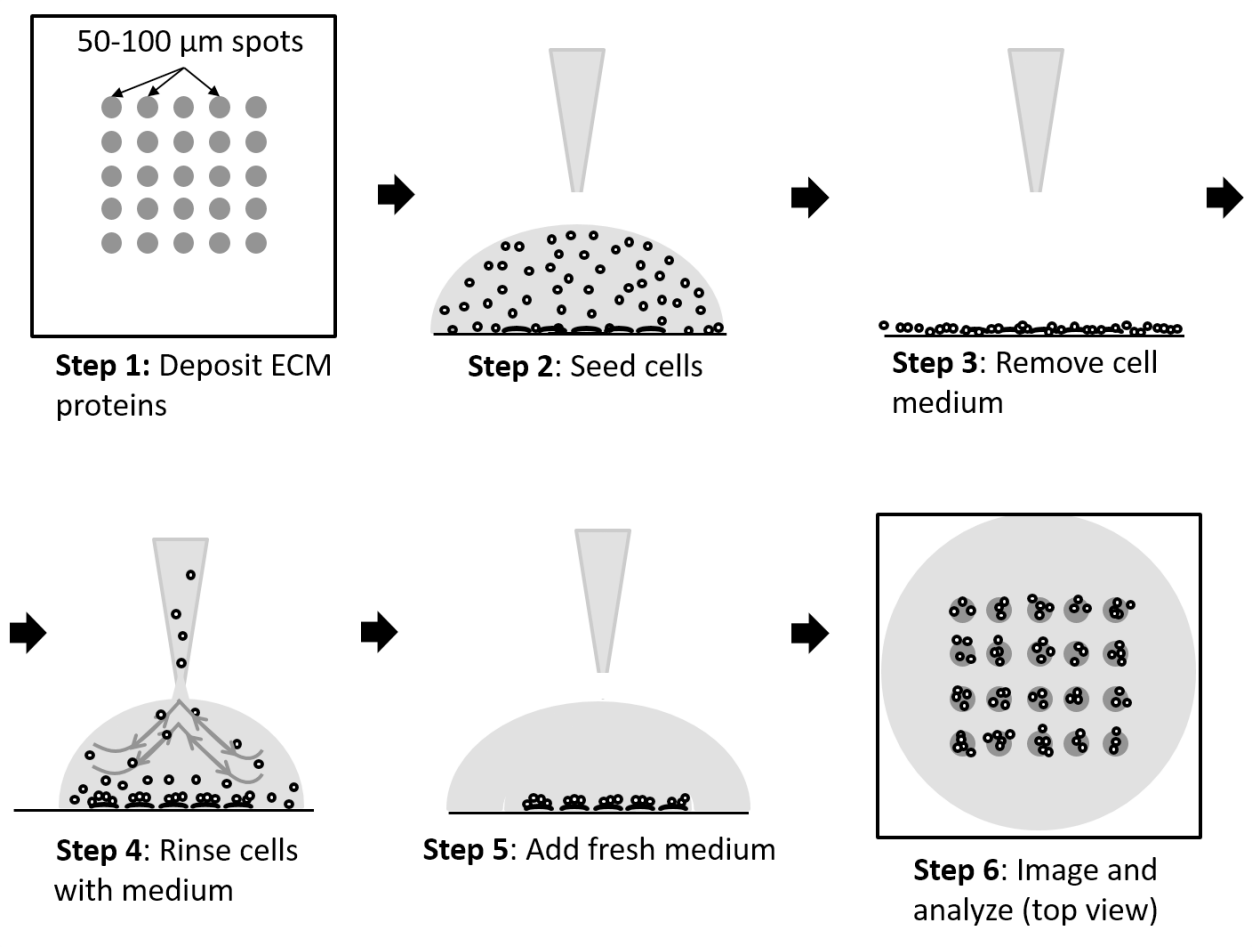
Cell seeding on patterned substrates workflow. Procedural steps for cell seeding and removal to produce spatially confined small populations (10–50 cells/population) of cells are shown.

**Fig 3:**
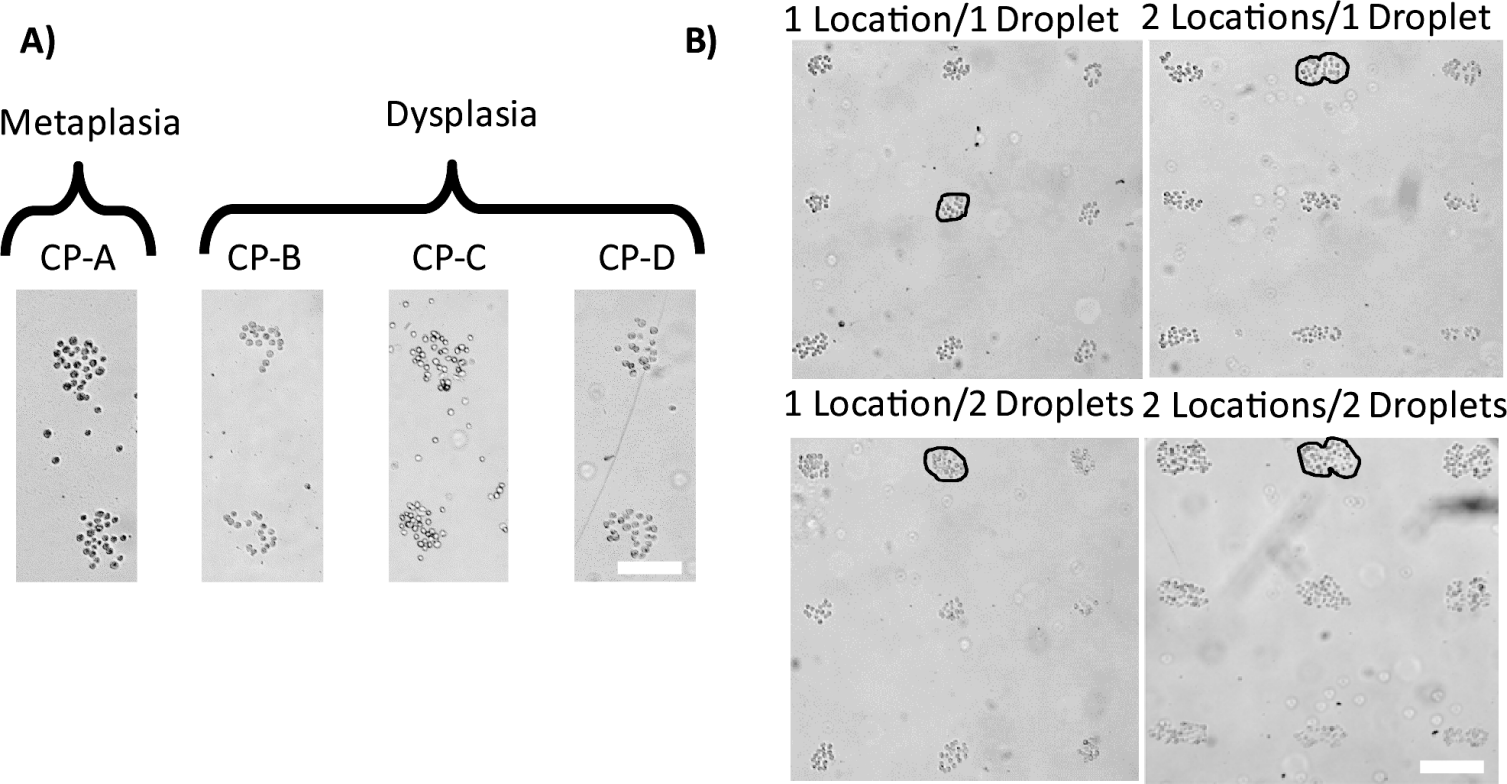
Patterning human esophageal epithelial cell types on substrates deposited with the ECM mixture. A) The four different cell types (CP-A, CP-B, CP-C, and CP-D) representing the metaplastic and dysplastic stages of premalignant BE progression showed similar population sizes and densities when seeded on custom ECM protein mixture. The micrographs were taken at 15 minutes after seeding. Scale bar 100 μm. B) Small populations of dysplastic (CP-D cell line) BE epithelial cells created using the four different patterning configurations detailed in Fig 4. Simple manipulations of the ECM mixture volume and the spatial arrangement of the deposition locations results in significantly different population sizes and numbers of cells per population. A circle has been drawn on the image to show that there is a difference in number between the cell populations. Custom deposition patterns with varying population sizes can be readily created by varying the distance between the spots and/or volume of the ECM mixture and its local deposition pattern. Scale bar 200 μm.

The workflow of obtaining cell populations on the patterned substrates consists of the following steps (Fig 2): 1. A small volume, typically 50 μL, of cell culture medium containing about 500 cells is pipetted onto the substrate; 2. Cells are allowed to settle via gravity and adhere to the patterned areas for 15 minutes; 3. The cell medium is removed; 4. Floating and weakly adhered cells outside of the patterned areas are removed via washing the substrate with fresh cell medium; 5. Fresh medium is added to the substrate; 6. Cells are imaged. Proof-of-principle of the approach was determined as follows. A mixture of extracellular matrix (ECM) proteins was used as the cell adhesion promoting material. The optimal formulation of the extracellular matrix (ECM) proteins mixture was obtained by varying the amounts of the ECM proteins, and seeding cells on patterned substrates. The seeded cells consisted of four different esophageal epithelial cell types representing the metaplasia (CP-A cell line) and dysplasia (CP-B, CP-C, CP-D cell lines) stages in premalignant progression in Barrett’s esophagus. The four cell lines of BE used in the study represent different stages (metaplasia to various sub-stages of dysplasia) of premalignant progression to esophageal adenocarcinoma with markedly different physiological, morphological, adhesion, and gene expression profiles [22, 23]. The main reason for choosing these four cell lines was to demonstrate the ability of our approach to pattern cells with different physiological and biomolecular (including adhesion) properties. The optimal ECM formulation was achieved when all four cell types showed almost identical results in terms of the patterned population size, density and spatial localization (Fig 3A). We note that ECM mixtures with different formulations can be made to accommodate differences in cell adhesion properties of other cell types.

**Fig 4:**
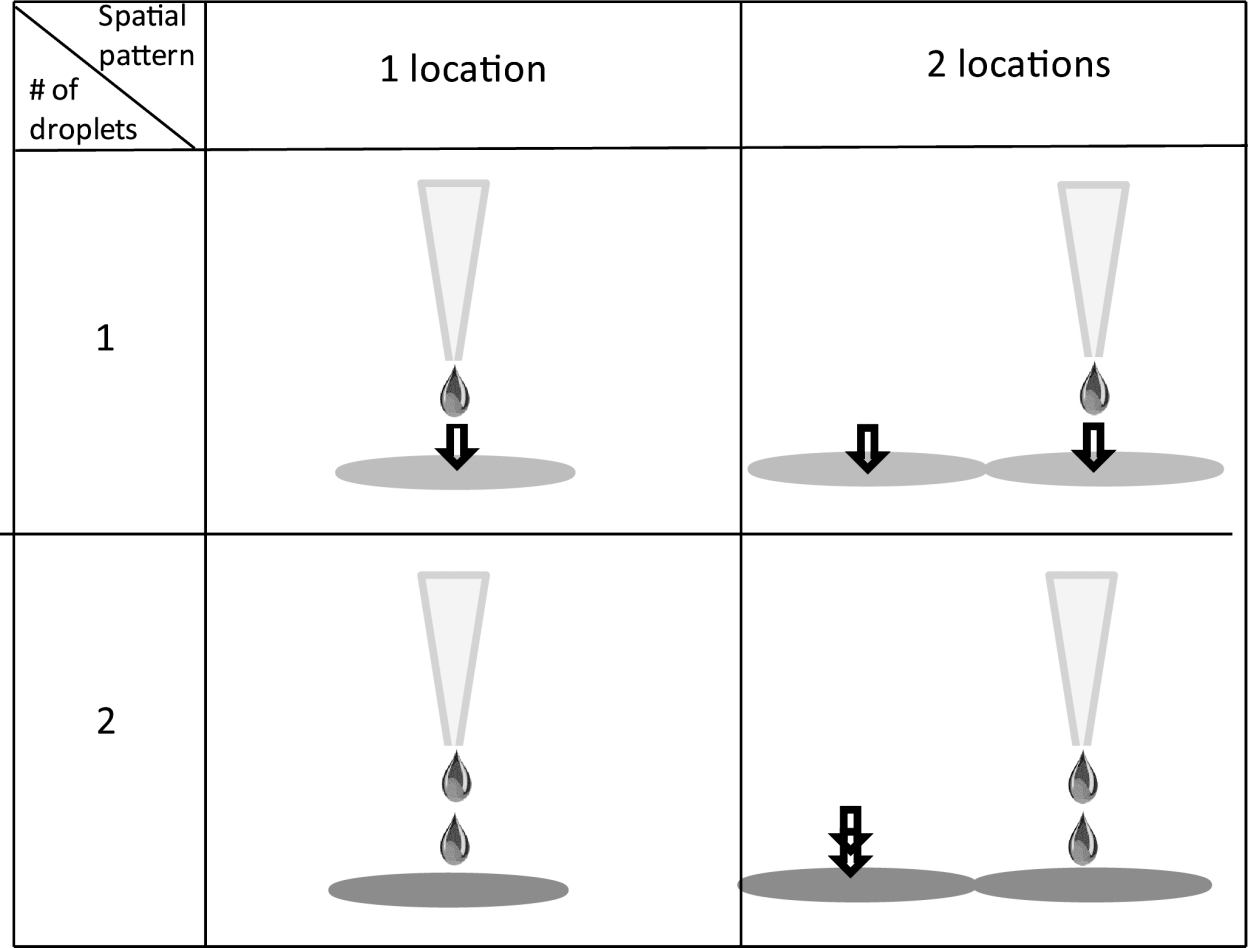
Spatial patterns of ECM mixture deposition used in this study to produce differently sized small populations of cells. The panels in the first row depict a pattern where one drop (~40 pL volume) of the ECM mixture was deposited on one (upper left) or two adjacent (upper right) locations on the substrate. The bottom two panels represent the same spatial patterns as above, but with two droplets of the mixture being deposited per location.

To characterize our approach in terms of how accurately one can control the cell population size, reproducibility and reliability, we have performed a series of experiments where we varied two parameters: the volume of the deposited ECM mixture and the surface area over which the ECM mixture was deposited. The CP-D cell line was used for the experiments. Our goal was to determine how precisely one can control the number of cells in a population as well as what are the limiting technical factors. A summary of the four different patterning designs for ECM deposition implemented in this study is shown in Fig 4. In the first experiment we deposited one droplet of the ECM mixture on one single spot. The second pattern was generated by dispensing one droplet on two adjacent spots. The third pattern was obtained by dispensing two droplets on the same spot, whereas the fourth configuration was accomplished via depositing two droplets on each of the two adjacent spots. These four patterns enabled exploration of the effect of varying functional area for cell adhesion as well as the amount of the ECM mixture deposited. Each deposition configuration was repeated 25 times (5x5 array) yielding a total of n = 25 populations for each pattern design. As expected, we observed distinct differences in the colony shape and size that grossly followed the different deposition patterns. The one drop/one location and two drops/one location patterns resulted in most cases in round colonies, whereas the colonies obtained using the two patterns with two locations were elongated with dumbbell-like shapes in some cases (Fig 3B). We note that the four studied cells types exhibited markedly different adhesion properties to the different ECM proteins. We found that the metaplastic BE (CP-A cell line) cell adhered preferentially to spots of the ECM proteins mixture used in the study (PLL+fibronectin+laminin), whereas almost no cells were observed adhered to the spots containing separately each one of the ECM proteins (S1 Fig). The CP-B and CP-D cells showed a preference of adhering to fibronectin, while the CP-C cells exhibited similar adhesion to fibronectin and the ECM mixture.

**Fig 5:**
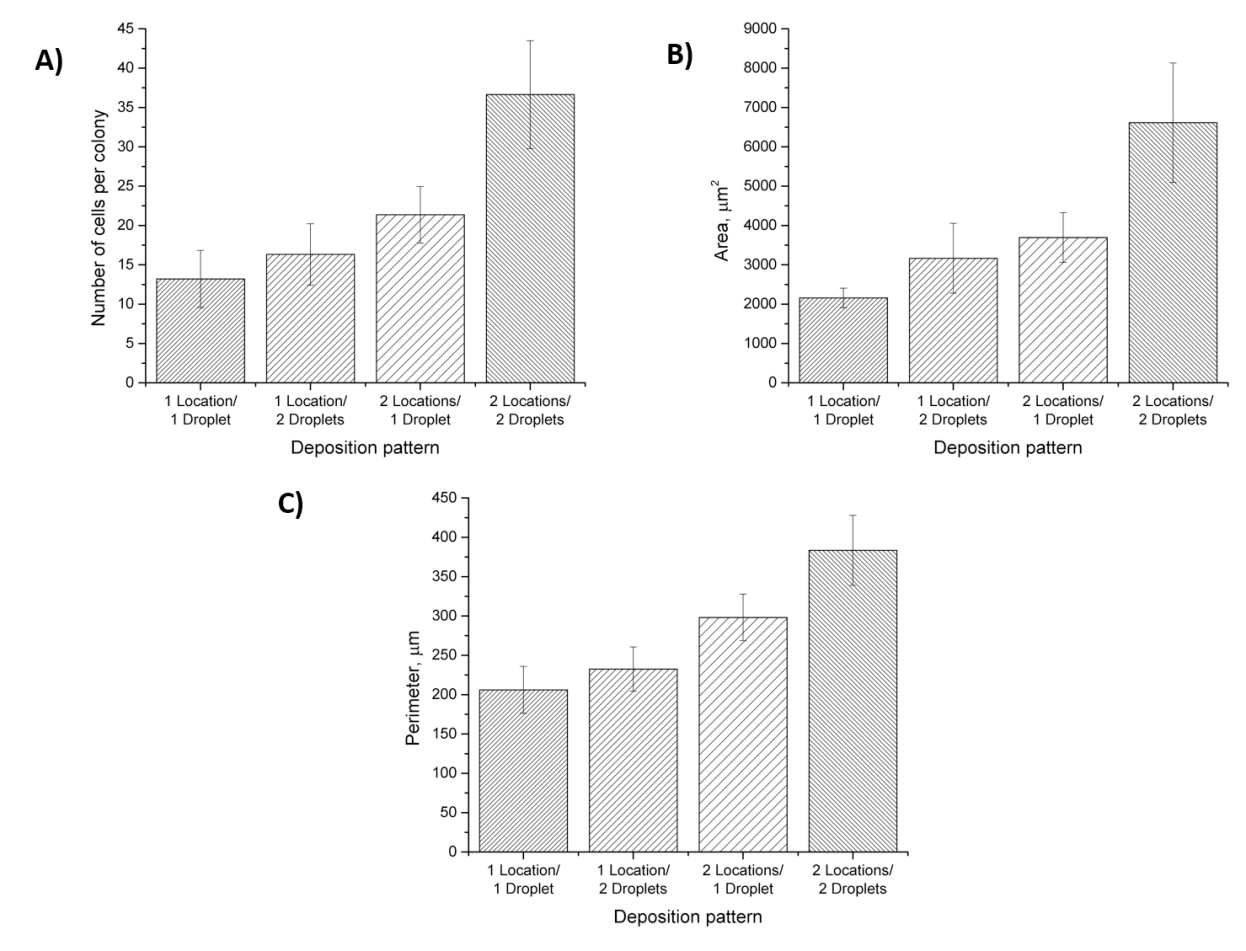
Characteristics of cell populations obtained under the four different deposition patterns shown in Fig 4. A) Average number of cells per population; B) Average surface area of populations; C) Average population perimeter. All three characteristics were significantly different (p = 0.05, two-way ANOVA, Tukey test)) among the two experimental parameters–number of drops and locations–indicating a robust performance and high level of control of the population size. Error bars represent standard deviation (SD).

The number of cells in each population was determined by manually counting the cells in brightfield micrographs. The average number of cells per population increased with the number of locations (spots) and/or droplets of the ECM mixture (Fig 5A). The average number of cells for the one location/one droplet configuration was 13.2±3.6 (SD), the average number of cells for the one location/two droplets was 16.3±3.9, the average number of cells for the two locations/one droplet was 21.4±3.6, and the average number of cells for the two locations/two droplets was 36.6±6.9 (Fig 5A). We found these results to be consistent across several trials and experimental days (Table 1). In addition to the number of cells per colony, we characterized the surface area and perimeter of the cell colonies obtained using the four different deposition patterns. The size parameters of the individual colonies were calculated by drawing a region of interest (ROI) around each of the colonies, such that the outline of the ROI coincided with the cells on the edge of the colonies. Similar to the number of cells, we observed a gradual increase in both area and perimeter with the number of locations or drops. The surface area of the colonies ranged from 2,160 ± 249 (SD) μm^2^ (one location/one droplet) to 6,600 ± 1,519 μm^2^ (two locations/two droplets), while the perimeter of the colonies increased from 206 ± 30 μm to 383 ± 44 μm for one location/one droplet and two locations/two droplets, respectively (Fig 5B and 5C, Table 1). A statistical significance test (two-way ANOVA, p = 0.05) showed that both patterning parameters–number of droplets and locations–yielded significantly different population sizes, surface area, and perimeter.

**Table 1:** Cell population parameters.

Deposition pattern	No. of cells/population				Surface area (μm^2^)				Perimeter (μm)			
	N	SD	CV	SEM	S	SD	CV	SEM	P	SD	CV	SEM
1 Location/1 Droplet	13.2	3.6	0.3	0.7	2,160	249	0.1	88	206	30	0.1	11
1 Location/2 Droplets	16.3	3.9	0.2	0.8	3,170	888	0.3	296	233	28	0.1	9
2 Locations/1 Droplet	21.4	3.6	0.2	0.7	3,700	631	0.2	210	298	30	0.1	10
2 Locations / 2 Droplets	36.6	6.9	0.2	1.4	6,600	1,519	0.2	506	383	44	0.1	15

N–average number of cells per population, S–average population surface area (μm^2^), P–average population perimeter (μm), SD–standard deviation, CV–coefficient of variation, SEM–standard error of mean.

**Fig 6:**
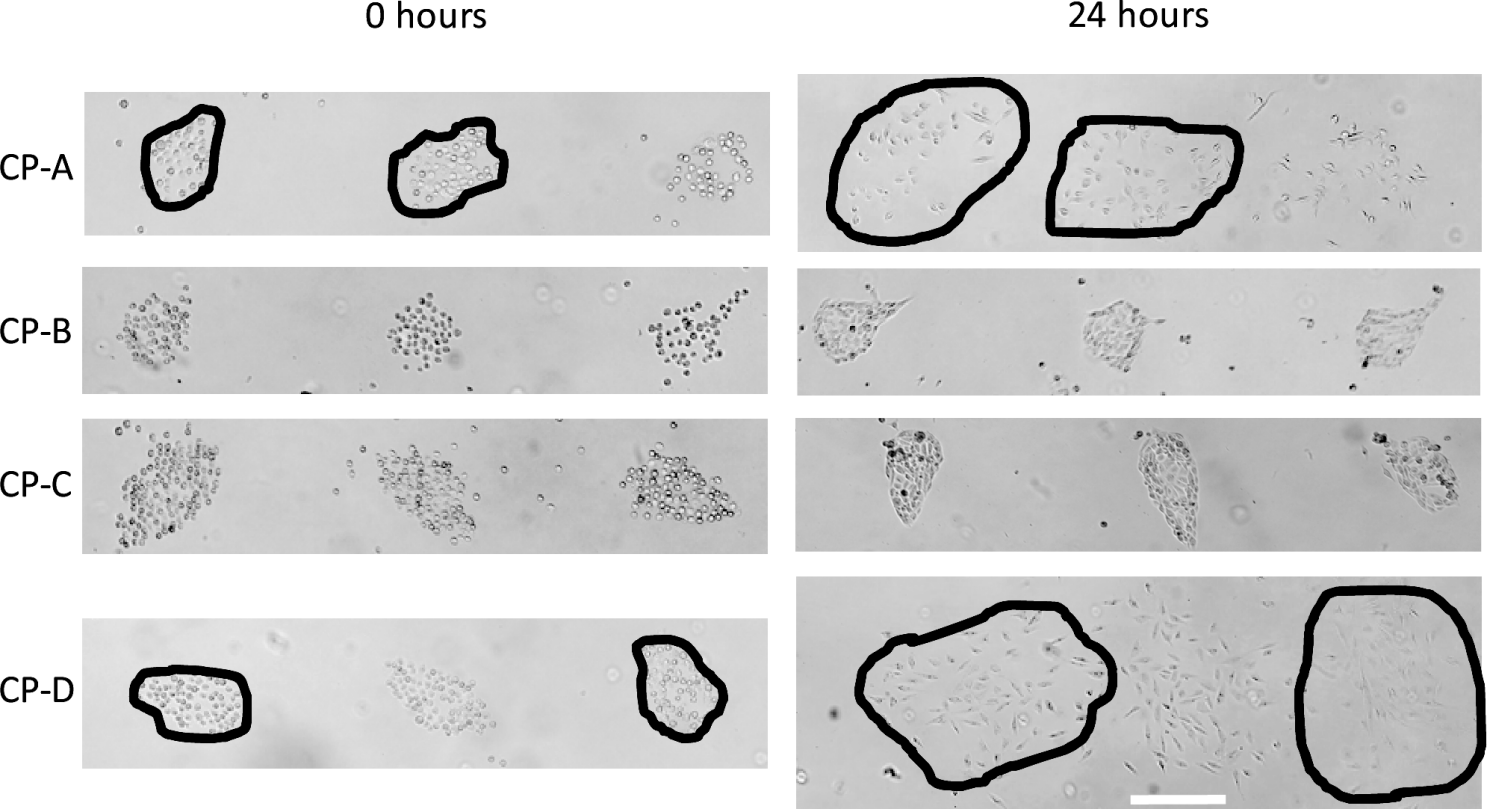
Behavior of cell populations over time. While two (dysplastic CB-B and CP-C) out of four studied cell types mostly retained the initial cell population size and shape, metaplastic CP-A and dysplastic CP-D cells exhibited more motile phenotype. The cells of the latter two cell types dispersed after 24 hours of incubation resulted in almost complete disappearance of the initial population shape. The ECM mixture was deposited using the two locations/two droplets deposition pattern. Scale bar 100 μm.

To assess the behavior of cells after seeding over time, we compared the morphology of the colonies immediately after seeding and at 24 hours after seeding (Fig 6). We observed that the colonies of dysplastic cells (CP-C and CP-B cell lines) mostly retained their initial shape over time. Changes in cell morphology from the circular to an elongated shape as well as increased density of the colonies indicate regular cell behavior that one usually observes in a petri dish. Cells of the other two cell types–CP-A and CP-D–showed a more motile behavior with strong migration patterns outside of the deposited spots of ECM and almost complete disappearance of the initial colony shape.

**Fig 7:**
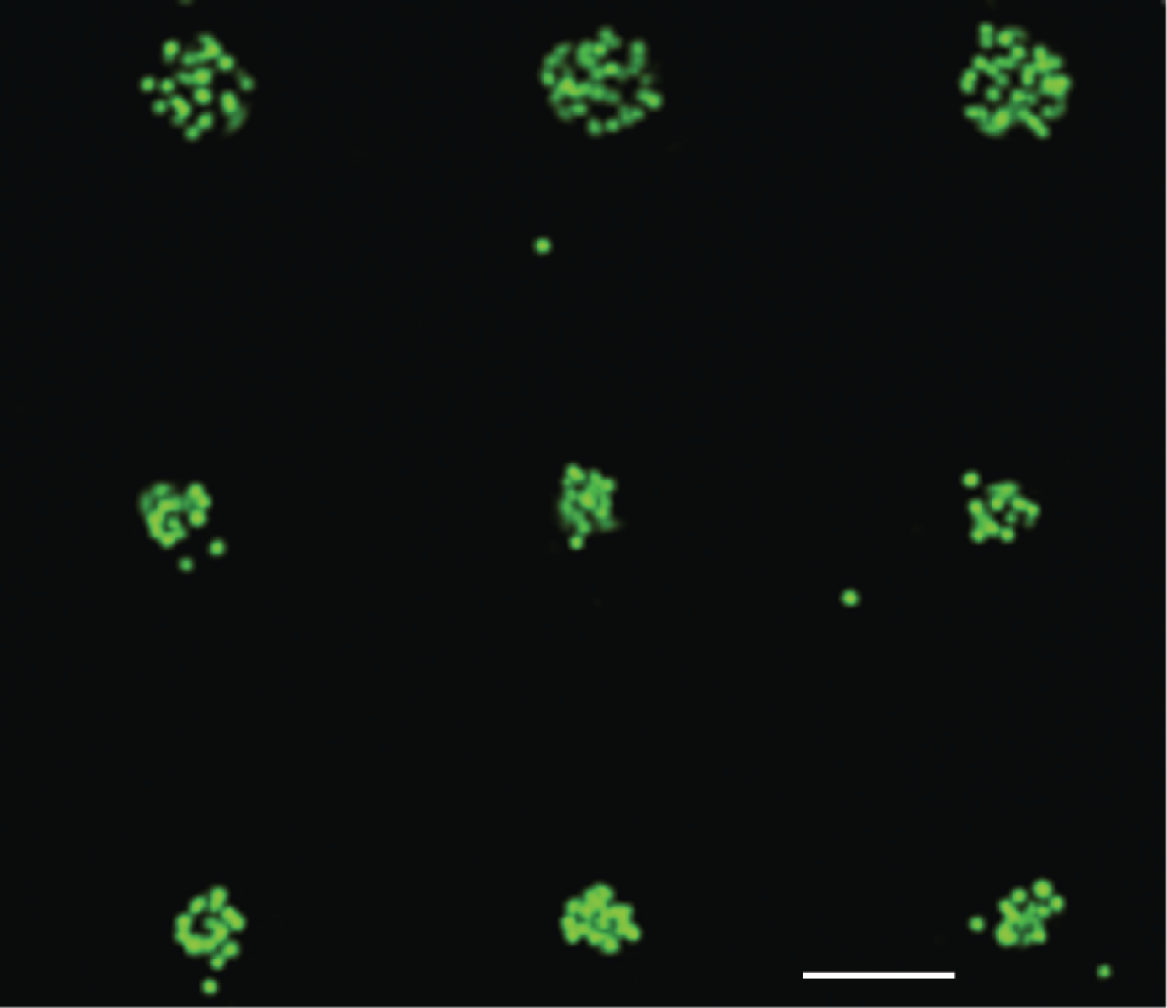
Cell viability on the patterned substrates after seeding. A fluorescence micrograph of a 3x3 array of ECM deposited spots after seeding and washing cells. The cells were stained with the LIVE/DEAD stain, where the green color indicates live cells and the red color dead cells. The green color was predominant in all viability assays, with only a few red (dead) cells appearing randomly and outside of the deposited regions. The overall cell viability was >99% (number of experiments n = 3). Scale bar 100μm.

Next, we determined cell viability after deposition on substrates. Using a live/dead fluorescence assay we found >99% cells (number of experiments n = 3) were alive after seeding on the patterned substrates (Fig 7) with only a few dead cells outside of the patterned regions. We determined that the growth rate of cells on patterned substrates at 24 hours after seeding was similar to cells grown in conventional cell culture dishes (data not shown). This result demonstrates that cell patterning using our approach does not induce significant stress on the cells and is amenable for live cell studies. Furthermore, the confinement of the cells within the patterned areas and almost complete absence of cells in the interstitial areas makes the approach amenable for cell signaling and migration studies, where one could observe and quantitate migration events of individual cells.

## Discussion

Our goal was to develop a method to reliably control the spatial organization of small populations of cells that could be simple, non-invasive, and widely accessible for a broad variety of biomedical research labs and did not require extensive training to perform. The ability to precisely control the volume of the deposited fluid and the droplet generation process via adjustable deposition parameters offered by the inkjet patterning platform enabled robust control of the colony size and shape. We demonstrated that generating small populations of cells (10–40 cells/population) on planar substrates can be performed with both high-throughput (~1 population/second) and precision (with 20–30% standard deviation) over the population size using differential cell adhesion agents patterned in custom spatial patterns. Small populations of premalignant metaplastic and dysplastic Barrett’s esophageal epithelial cells were successfully generated in a size range of 13–37 cells by simply varying the volume and surface area of the adhesion-promoting regions followed by randomly seeding cells on these patterns and then rinsing with PBS. We note that the four studied cell types exhibited different adhesion preferences. For example, metaplastic (CP-A) cells adhering to the ECM mixture, but not to each one of the ECM components, whereas dysplastic (CP-D) cells showed preference to adhere to the fibronectin spots. While we did not study this interesting observation any further, it is conceivable that the marked differences in gene expression levels between the metaplastic (CP-A) and dysplastic (CP-B, -C, and–D) cells, may manifest also in an altered expression profile of the membrane proteins and/or their post-translational modifications, which may be responsible for the observed difference in the adhesion preferences. More detailed studies would be needed to confirm this assumption. Due to the ability to adjust the formulation of the ECM proteins mixture, we were able to pattern all four cell types using a single mixture with comparable results.

The method does not require photolithography or clean room facilities, thus making it accessible to a wide range of laboratories. By using the Rainmaker platform, we were able to create uniform arrays of areas coated with ECM proteins that served as facilitated adhesion locations for the cells. While we patterned the substrates with an ECM mixture via deposition with a high-end fluidic dispensing platform, any other approaches amenable for deposition of small (0.1–1 nL) fluid volumes, e.g. ink-jet printing based methods, can be employed following the same principle. Depositing the ECM mixture in small quantities eliminates the need for micro-patterning methods that require advanced instrumentation and skill to implement. Depending on how small the dispensed volumes are, different sizes of cell populations can be created on the substrates with adequate precision. In our study, the size of the patterns varied. These areas could be increased or decreased accordingly by altering the number of droplets and the surface area over which the droplets were deposited. Moreover, one can vary the formulation of the adhesion-promoting agent as an additional experimental parameter to achieve a higher level of control over the number and/or surface area of the cell population. It is, for example, conceivable that by lowering the viscosity of the adhesion agent solution, one can obtain larger spots via improved material wetting of the substrate. Furthermore, altering surface properties of the substrate via targeted functionalization to increase or decrease, for example, hydrophobicity would provide another parameter for more precise control over population size and its spatial characteristics.

## Conclusions

The presented method for patterning ECM proteins combines a suite of desirable experimental characteristics, including high level of control over cell number per population, population size (surface area), shape, and spatial location while being of low complexity and minimally invasive. These properties facilitate the wide use of the approach in biomedical research labs. Patterned substrates may be made applicable to a wide variety of cells and media determinant on further studies by controlling liquid deposition parameters. In contrast to other published inkjet printing approaches for cell patterning, our method provides the advantage of manipulating the formulation of the deposited adhesion solution (relative amounts of ECM proteins) potentially enabling the patterning of a broader variety of cell types with differing adhesion and growth preferences. The method is capable to create tunable spatial patterns to trap cells into a particular area, vary the amount of cells, and the population area if desired. A high level of reproducibility of cell numbers and cell populations deposited to the desired areas lend themselves for use in cell-to-cell communication, motility, and microenvironmental studies. Future work will be directed towards extending the utility of the method to 3D printing of complex tissue-like structures.

## Supporting information

S1 Fig Adhesion properties of the four BE cell types. Differential adhesion of the four studied cell types to the used ECM proteins and their mixture. The formulation of the mixture was the same as used in the rest of the study (Materials and methods).
(TIF)
