# Supplementary figures and images for: A versatile method for dynamically controlled patterning of small populations of epithelial cells on substrates via non-contact piezoelectric inkjet printing
Source: PLoS One. 2017 Apr 26;12(4):e0176079. doi: 10.1371/journal.pone.0176079 (PMC5406020; doi:10.1371/journal.pone.0176079)

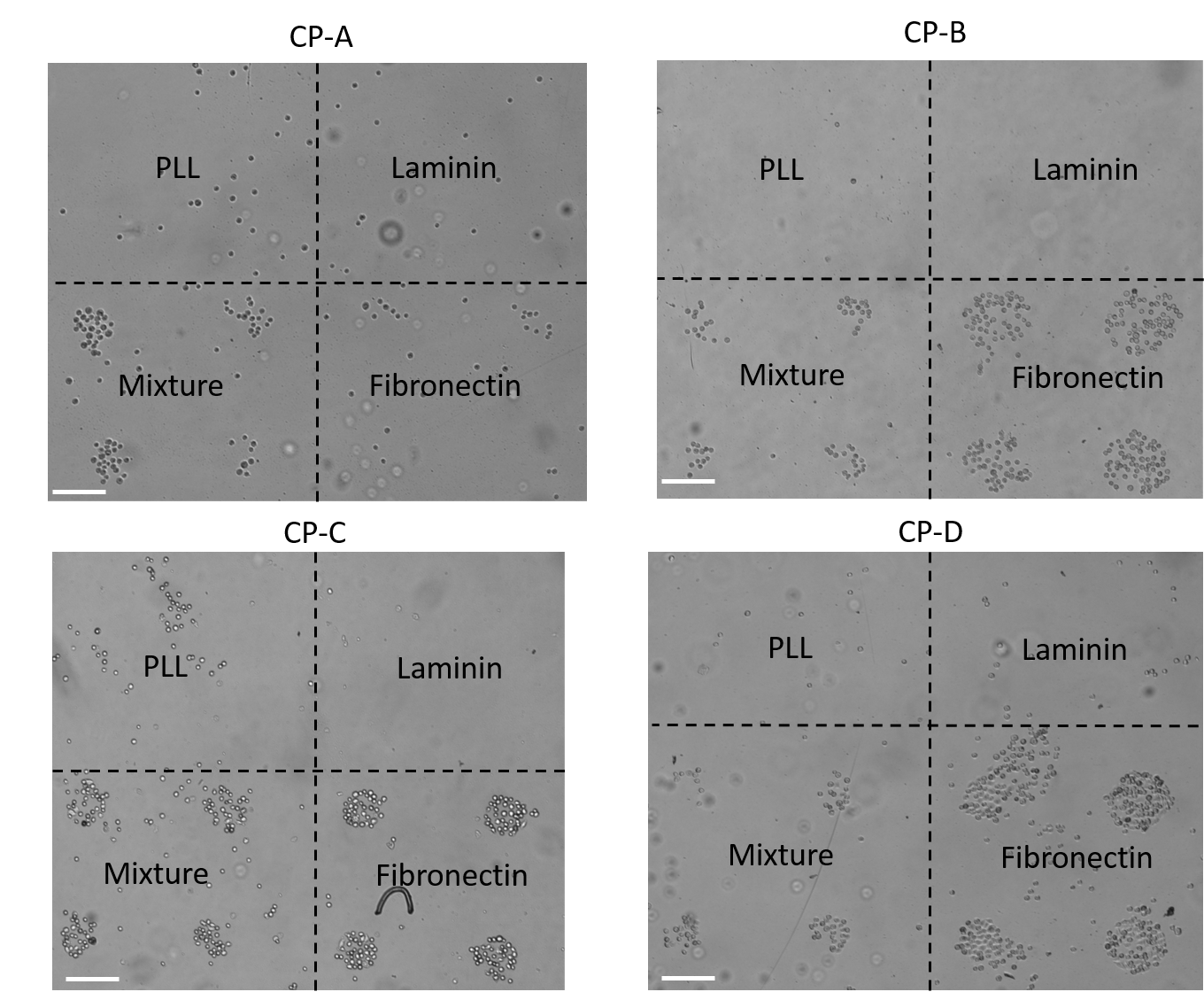

Supplement: S1 Fig — Differential adhesion of the four studied cell types to the used ECM proteins and their mixture. The formulation of the mixture was the same as used in the rest of the study (Materials and methods). (TIF) [file pone.0176079.s001.tif]
